# Eliciting cytotoxic T lymphocytes against human laryngeal cancer-derived antigens: evaluation of dendritic cells pulsed with a heat-treated tumor lysate and other antigen-loading strategies for dendritic-cell-based vaccination

**DOI:** 10.1186/s13046-016-0295-1

**Published:** 2016-01-22

**Authors:** Fan-Qin Wei, Wei Sun, Thian-Sze Wong, Wei Gao, Yi-Hui Wen, Jia-Wei Wei, Yi Wei, Wei-Ping Wen

**Affiliations:** Department of Otorhinolaryngology Head and Neck Surgery, the First Affiliated Hospital of Sun Yat-sen University, 2nd Zhongshan Road 58#, Guangzhou, 510080 Guangdong P.R. China; Institute of Otorhinolaryngology Head and Neck Surgery, Sun Yat-sen University, 2nd Zhongshan Road 58#, Guangzhou, 510080 Guangdong P.R. China; Department of Otorhinolaryngology Head and Neck Surgery, the Sixth Affiliated Hospital of Sun Yat-Sen University, Yuancun Second Cross Road 26#, Guangzhou, 510655 Guangdong P.R. China; Department of Surgery, The University of Hong Kong, Pokfulam Road 102#, Hong Kong, P.R. China

**Keywords:** Antigen loading, Antigen presentation, Dendritic cells, Tumor immunity, Laryngeal cancer, Tumor lysate

## Abstract

**Background:**

Dendritic cells (DCs) have been used successfully in clinical pilot studies. However, tumor-specific immunity and clinical responses were only induced in certain cancer patients. It has been well documented that immunotherapy efficacy can be optimized for responses using antigen pulsing.

**Methods:**

The human laryngeal squamous cell cancer (LSCC) cell line SNU899 was used to evaluate the in vitro anti-tumor efficacy of three different preparations of dendritic cell (DC) vaccines consisting of either whole tumor cells or their derivatives including: i) DCs pulsed with a tumor cell supernatant (DC-TCS), ii) DCs pulsed with whole-cell tumor stressed lysate (DC-TSL), and iii) DCs pulsed with irradiated tumor cells (DC-ITC).

**Results:**

Our results showed that DC-TSL is an effective source of tumor-associated antigens (TAAs) for pulsing DCs. DC-TSL induced the highest expansion of TAA-specific T cells, the strongest Th1 cytokine response, and the most potent cytotoxic T lymphocyte (CTL) activity. DC-TCS and DC-ITC inhibited T cell activation but induced a certain extent of CTL activity.

**Conclusions:**

These data suggest that DC-TSL is a more potent inducer of antitumor immunity against laryngeal cancer than other antigen-loading strategies using whole tumor cell materials. This strategy provides an alternative approach for DC-based immunotherapy for laryngeal cancer.

## Background

Each year, more than 500,000 cases of head and neck squamous cell carcinoma (HNSCC) are diagnosed worldwide [1]. Laryngeal squamous cell carcinoma (LSCC) accounts for a large proportion of HNSCCs [2]. Despite the rapid development of diagnosis and treatment of LSCC, including surgery, chemotherapy, and radiotherapy, the 5-year overall survival rate of LSCC remains relatively low [3]. The high rate of local recurrence and considerable mortality that accompany this malignancy mandate the development of novel therapeutic alternatives.

Immunotherapy is regarded as a promising strategy that complements current cancer therapies [4]. Dendritic cell (DC)-based vaccination trials have been performed for some advanced cancers, such as melanoma and colon, renal, and prostate cancers [5–7]. They have proven to be feasible and safe, and to elicit immunological responses [8]. Current vaccination strategies include antigen (Ag)-defined vaccines (peptide, DNA, and recombinant tumor proteins) and polyvalent undefined vaccines (tumor lysate, heat shock proteins (HSPs), whole tumor cells, and whole tumor RNA) [5,6]. The former type of vaccine is prepared using a single tumor-associated antigen (TAA). However, its application using specific peptides is limited to patients expressing tumor-associated MHC class I restricted peptides that fail to elicit MHC class II and CD40L-mediated CD4+ T cell helper responses. In contrast, the latter type of vaccine, generated using whole tumor cells or their derivatives, are advantageous in that they provide a full complement of TAAs, including both MHC class I and class II-restricted epitopes, thus reducing the risk of immune escape by antigen loss variants.

Whole tumor cell Ag-loading strategies for DC-based vaccination have commonly used freeze-thawed tumor cell lysates as an immunogenic source of TAAs [9, 10]. However, DCs loaded with such lysates have provided only modest protection in various animal models [9, 11, 12] and stimulated limited responses in a range of clinical trials [10, 13, 14]. Some studies have even shown that freeze-thawed lysates suppress DC maturation and functions in vitro, and are an ineffective loading strategy for DC therapy in vivo [15, 16].

To enhance antitumor immunity, studies have attempted to optimize antigen loading. Compared with ordinary lysates obtained by subjecting tumor cells to several freeze (liquid nitrogen) and thaw (37 °C water bath) cycles, stressed lysates prepared from heat-treated (≥42 °C) tumor cells are a more effective source of TAAs for pulsing DCs. Heat-treated tumor lysate-pulsed DCs generate a stronger and broader T cell response against pancreatic cancer [17]. Irradiated tumor-primed DCs provide more potent protection against solid tumors, such as human melanoma [15]. Even retinoblastoma cell supernatants exert an immunostimulatory effect on DCs [18]. Because different tumor strains have various immunogenicities, it remains uncertain which Ag-loading strategy has superior efficacy for treating human laryngeal carcinoma.

In this study, we performed a detailed examination of DC phenotypes loaded with TAAs, including a tumor supernatant (TCS), tumor-stressed lysate (TSL), and irradiated tumor cells (ITC). Subsequently, we assessed TAA-specific T cell proliferation and Th1 cytokine production induced by these Ag-loaded DCs. Lastly, we compared the in vitro cytotoxic T lymphocyte (CTL) responses. Our study identified the human LSCC cell line SNU899-derived TSL as an efficacious source of TAAs for pulsing DCs to induce a more potent CTL response against laryngeal cancer.

## Methods

### LSCC cell line

SNU899 cells were kindly provided by Professor Ja-Lok Ku (Seoul National University College of Medicine, South Korea). Cells were maintained in RPMI-1640 containing 10 % fetal bovine serum, 100 U/ml penicillin, and 100 μg/ml streptomycin (Sigma, St. Louis, MO, USA) in a humidified incubator at 37 °C with 5 % CO_2_.

### Ethics statements

The study protocol (No. 2012–349) was approved by The First Affiliated Hospital of Sun Yat-sen University Ethics Committee, and was used for research purposes only. Donor informed consent was obtained before enrollment.

### DCs

Peripheral blood mononuclear cells (PBMCs) were isolated from healthy individuals by Ficoll-Hypaque density gradient centrifugation. CD14^+^ cells were purified using a magnetic bead-conjugated mouse anti-human CD14 monoclonal antibody (CD14 MicroBeads, Miltenyi Biotec, Bergisch Gladbach, Germany). The purity of CD14^+^ cells was consistently more than 95 %. Cells were seeded into six-well plates at 1.5 × 10^6^ cells/well in 2.5 ml RPMI-1640 medium supplemented with 10 % heat-inactivated FBS, 50 ng/ml granulocyte macrophage-colony stimulating factor (GM-CSF, PeproTech, Rocky Hill, NJ, USA), 20 ng/ml interleukin (IL)-4 (R&D Systems, Minneapolis, MN, USA), 100 U/ml penicillin, and 100 μg/ml streptomycin, and incubated at 37 °C in a humidified atmosphere with 5 % CO_2_. Half-volume medium changes were performed every 3 days. On day 7, DC maturation was induced by addition of 1 μg/ml lipopolysaccharide (LPS, Sigma) for an additional 2 days for in vitro experiments. DC phenotypes were determined by flow cytometry.

### DC loading with SNU899 LSCC-derived Ags

Day 6 DC cultures were loaded with SNU899-derived Ags using three methods. (1) Pulsing with a tumor cell supernatant prepared by seeding 4.5 × 10^6^ cells in a T75 flask (Eppendorf, Hamburg, Germany) containing 4.5 mL completed medium for 24 h, followed by centrifugation to remove cell debris. (2) Pulsing with a whole-cell TSL prepared at a concentration of 4.5 × 10^6^ cells/well in 0.5 ml RPMI-1640 medium and heat-treated at 42 °C and 60 °C in sequence for 1 h each in a thermostatically controlled water bath, followed by immediate freezing in liquid nitrogen for 10 min and thawing at room temperature. This procedure was repeated four times. Cell debris were removed by centrifugation at 400 × *g* for 20 min. (3) Pulsing with ITC prepared at a concentration of 4.5 × 10^6^ cells/well in 0.5 ml RPMI-1640 medium and subjected to 1 × 10^4^ Rads of irradiation [15]. All methods employed a tumor:DC ratio of 3:1 and incubation at 37 °C for 24 h.

### T cell priming by Ag-loaded autologous DCs

Frozen PBMCs were thawed, resuspended in complete medium, and cultured overnight in a T25 flask (Eppendorf). Peripheral blood lymphocytes (PBLs) were partially purified by negative depletion from the nonadherent fraction of PBMCs after removal of monocytes by adhesion to the culture flask. PBLs were seeded in a round-bottom 96-well plate at 2 × 10^5^ cells/well. The three different Ag-loaded DC preparations were added to autologous PBLs at a ratio of 1:20. After 1 week, a second identical stimulation was performed. Half of the medium was replaced with fresh medium containing 20 U/ml IL-2 twice per week. All experiments were performed in triplicate. PBLs alone were used as a control. The cultures were incubated at 37 °C with 5 % CO_2_. CD4^+^ and CD8^+^ T cell proliferation and intracellular cytokine production in CD4^+^ T cells were assessed by flow cytometry on day 6 after the second stimulation by surface and intracellular staining.

### In vitro induction of TAA-specific CTL responses by tumor-derived Ag-loaded DCs

The Ag-loaded DCs prepared by different methods were compared for their ability to stimulate CTL responses. After Ag loading and maturation, the DCs (stimulators) were added to PBLs (autologous responders to the DCs) at a ratio of 1:20 in a round bottom 96-well plate. Unpulsed mature DCs were used as a control. After 1 week, a second identical stimulation was performed. Half of the medium was replaced with fresh medium containing 20 U/ml IL-2, twice per week. On day 6, PBLs were harvested and assessed for CTL activity. The targets used for the CTL assay were SNU899-derived lysate-pulsed immature DCs autologous to the CTLs. These DC were not mature, unlike those used for CTL stimulation, because immature Ag-pulsed DCs are susceptible to CTL-mediated killing, whereas mature DCs are protected from lysis [19].

For CTL assays, targets were labeled with 5 μM 5,6-carboxyfluorescein diacetate succinimidyl ester (eBioscience, San Diego, CA, USA) for 10 min in the dark at room temperature, and applied at an effector:target (E:T) ratio of 10:1 using 2 × 10^4^ target cells/well in a round-bottom 96-well plate. In parallel, target cells were incubated alone to measure basal apoptosis. Cells were incubated for 6 h at 37 °C with 5 % CO_2_. Cytotoxicity was assessed by flow cytometry with annexin V and 7-aminoactinomycin D (7-AAD) staining [20].

### Flow cytometry and antibodies

DC phenotypes were determined using the following anti-human monoclonal antibodies: anti-CD1a-PE-Cy7, anti-CD83-FITC, anti-HLA-DR-eFluor 450, anti-CD80-PE-Cy5, anti-CD86-PE, and anti-CD40-APC. On day 6, PBLs were harvested and stained with the following anti-human monoclonal antibodies: anti-CD3-eFluor 450, anti-CD4-FITC, and anti-CD8a- PE-Cy7 for surface staining; anti-interferon (IFN)-γ-APC-eFluor780, anti-IL-2-PE-Cy7, and anti-tumor necrosis factor (TNF)-α-Alexa Fluor 700 for intracellular staining. Soluble anti-CD3 (OKT3, 0.5 μg/ml) and anti-CD28 (CD28.2, 2 μg/ml) monoclonal antibodies were used for in vitro activation of T cells. All antibodies and isotype controls were purchased from eBioscience. Samples were analyzed using a flow cytometer (LSRFortessa, BD, Franklin Lakes, NJ, USA). To examine apoptosis, target DCs were stained with APC-annexin V and 7-AAD (BD), and analyzed using a FACSCantoII flow cytometer (BD). Data were processed using the accompanying software (FACSDiva, BD).

### Statistical analysis

Experiments were repeated at least twice. Statistical analysis was carried out using SPSS version 13.0 software (IBM, Chicago, IL, USA) for Windows. Data are expressed as means and standard deviation (SD). Differences between the means were compared using Student’s t-test. A difference between two variables was considered significant when the two-tailed *P* value < 0.05 (95 % confidence level).

## Results

### Effect of different Ag-loading strategies on DCs

**Fig. 1:**
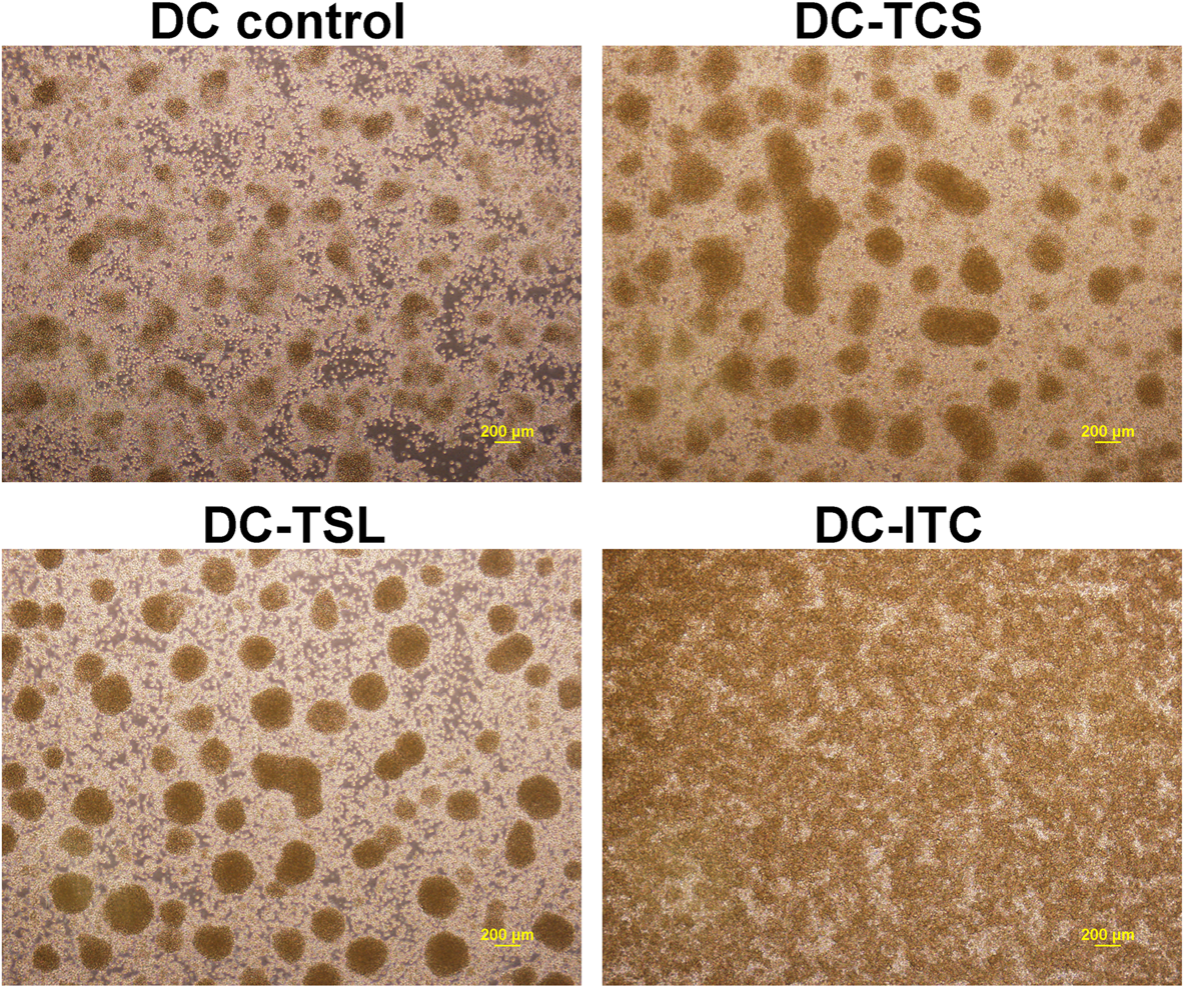
Photomicrographs of DC cultures (4×). DCs cultured for 5 days were pulsed for 24 h with SNU899 Ags using three different methods. All methods employed a tumor:DC ratio of 3:1. Compared with the DC control (≤200 μm), DC-TCS or DC-TSLs appeared to form larger adherent aggregates (>200 μm), whereas DC-ITC had a higher density

**Fig. 2:**
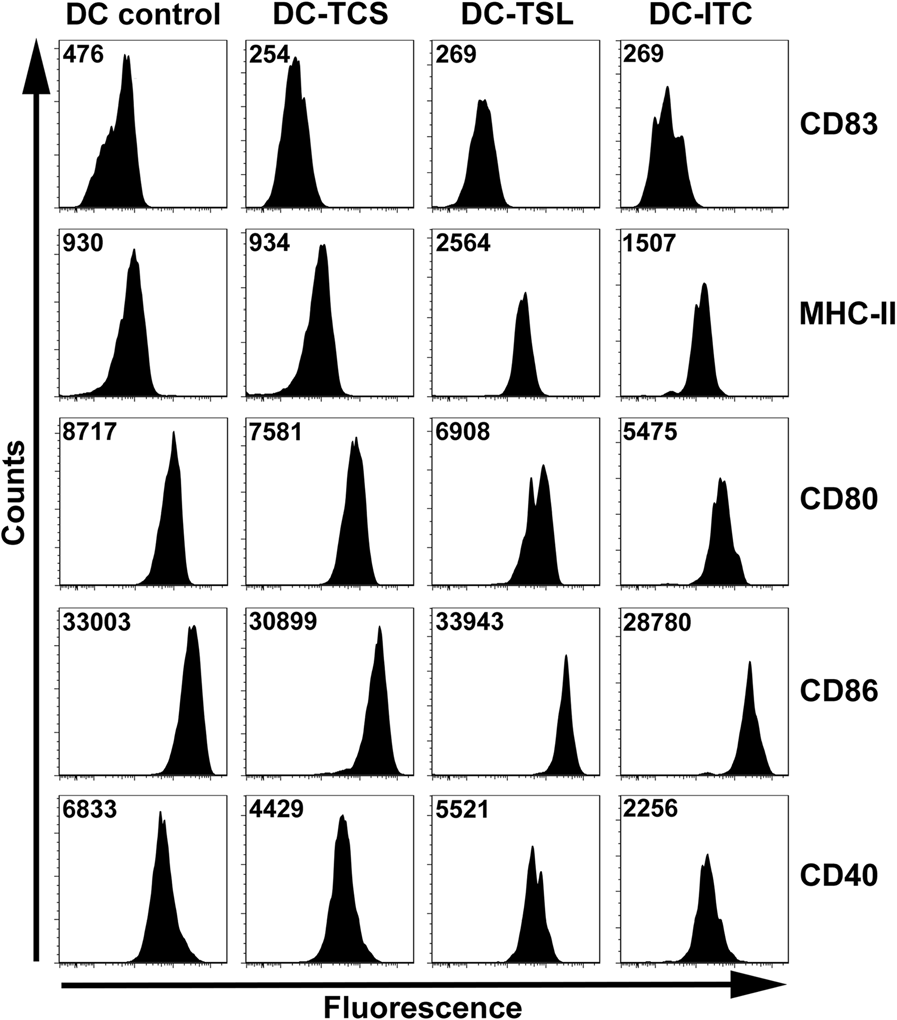
Phenotypes of DCs loaded with SNU899-derived Ags by different methods. To induce maturation, control DCs and SNU899-derived Ag-loaded DCs were treated with 1 μg/ml LPS for 48 h. A DC maturation marker (CD83), MHC-II molecule (HLA-DR), and co-stimulatory molecules (CD80, CD86, and CD40) were measured by flow cytometry. DC-TSL expressed the highest level of HLA-DR and CD86. DC-ITC also exhibited elevated HLA-DR expression, but expressed the lowest level of co-stimulatory molecules. HLA-DR expression on the surface of DC-TCSs remained at the same level as in unpulsed DCs. Data shown are representative of three experiments

CD14^+^ cells were purified from PBMCs using CD14 MicroBeads. Upon treatment with GM-CSF and IL-4, the majority of cells formed clusters, displayed typical dendritic morphology, and became CD1a^+^ (94.1–99.7 %), which are indicative of a DC phenotype. On day 6, DCs were treated for 24 h with SNU899-derived Ags using three strategies. Compared with control DCs, DC-TCS or DC-TSL formed larger and more aggregates, whereas the DC cell density was relatively high after co-culturing with ITC (Fig. 1). DC maturation was induced on day 7 by treatment with 1 μg/ml LPS for 2 days, and evaluated by flow cytometry (Fig. 2). DC-TSL expressed the highest levels of HLA-DR and CD86. DC-ITC also had elevated HLA-DR expression, but expressed the lowest levels of co-stimulatory molecules (CD80, CD86, and CD40). HLA-DR expression on the surface of DC-TCS remained at the same level as that in DC controls.

These data suggest that TSLs and ITC may provide much more Ags than SNU899 culture supernatant for presentation by DCs. Furthermore, tumor cell Ags may affect DC activation in response to maturation signals such as LPS. Interestingly, tumor Ags promoted cluster formation and thus may contribute to DC proliferation.

### Induction of TAA-specific T cell responses by Ag-loaded DCs

**Fig. 3:**
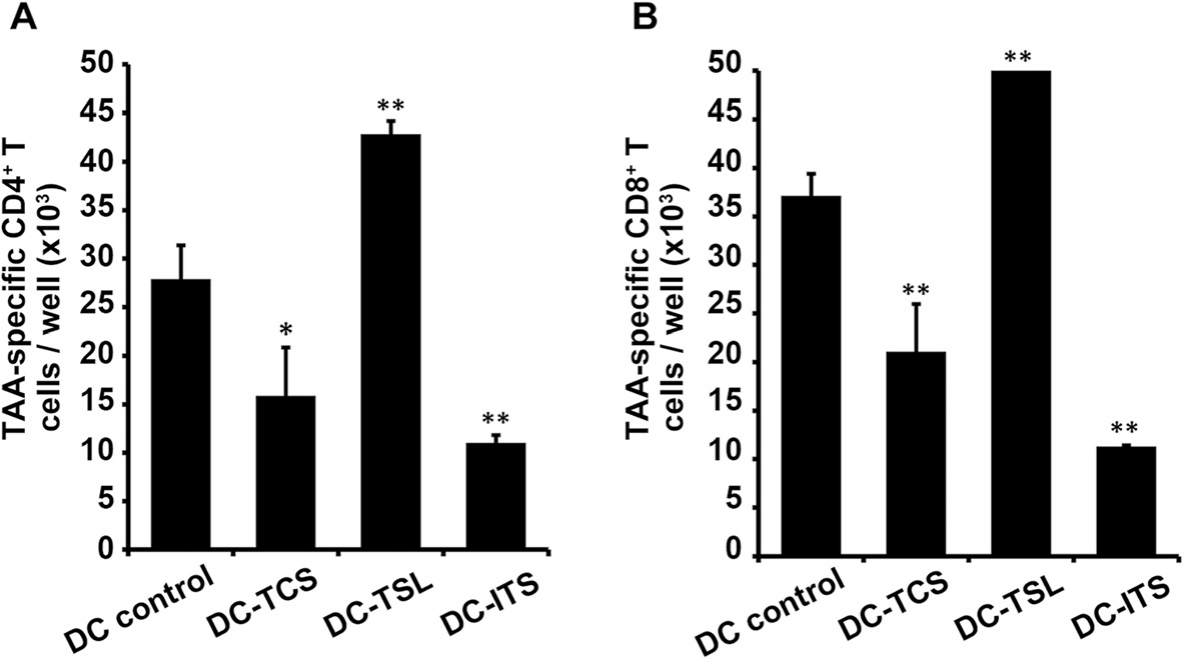
Induction of TAA-specific T cell responses by DCs loaded with SNU899-derived Ags by various methods. **a** Induction of TAA-specific CD4^+^ T cells. TAA-loaded mature DCs were added to autologous PBLs at a ratio of 1:20. On day 6 after the second stimulation, the number of CD4^+^ T cells was assessed by flow cytometry. The methods used to load the DCs induced differential class II cross-presentation of TAAs to the T cells. However, the stressed lysis strategy resulted in the highest efficiency of T cell activation, reflecting a higher level of TAAs after loading. **b** Induction of TAA-specific CD8^+^ T cells. Similar to the cross-presentation by MHC class II, TCL-DCs were also much more efficient at cross-presentation of TAAs by MHC-I, and thus T cell activation, than DC-TCS or DC-ITC. Data are mean values ± SD of triplicate determinations. Statistical comparisons were performed using Student’s t-test. *P < 0.05 vs. DC control; **P < 0.01 vs. DC control

Generation of TAA-specific T cells by the three DC-loading strategies were compared by flow cytometry after two stimulations with Ag-loaded mature DCs. Induction of TAA-specific T cells by Ag-loaded DCs was dependent on the Ag-priming strategy, as indicated by the increased numbers of CD4^+^(Fig. 3a) and CD8^+^ (Fig. 3b) induced by DC-TSL, compared with DC-TCS or DC-ITC. This result strongly suggests that DC loading induces differential MHC class I and II cross-presentation of TAAs to T cells. However, this observation differs from previous reports [15, 21]. The stressed lysis strategy of the tumor cell line SNU899 resulted in the highest efficiency of T cell activation, which reflected the higher levels of TAAs after loading.

### Analysis of TAA-specific Th1 cytokine response induced by Ag-loaded DCs

**Fig. 4:**
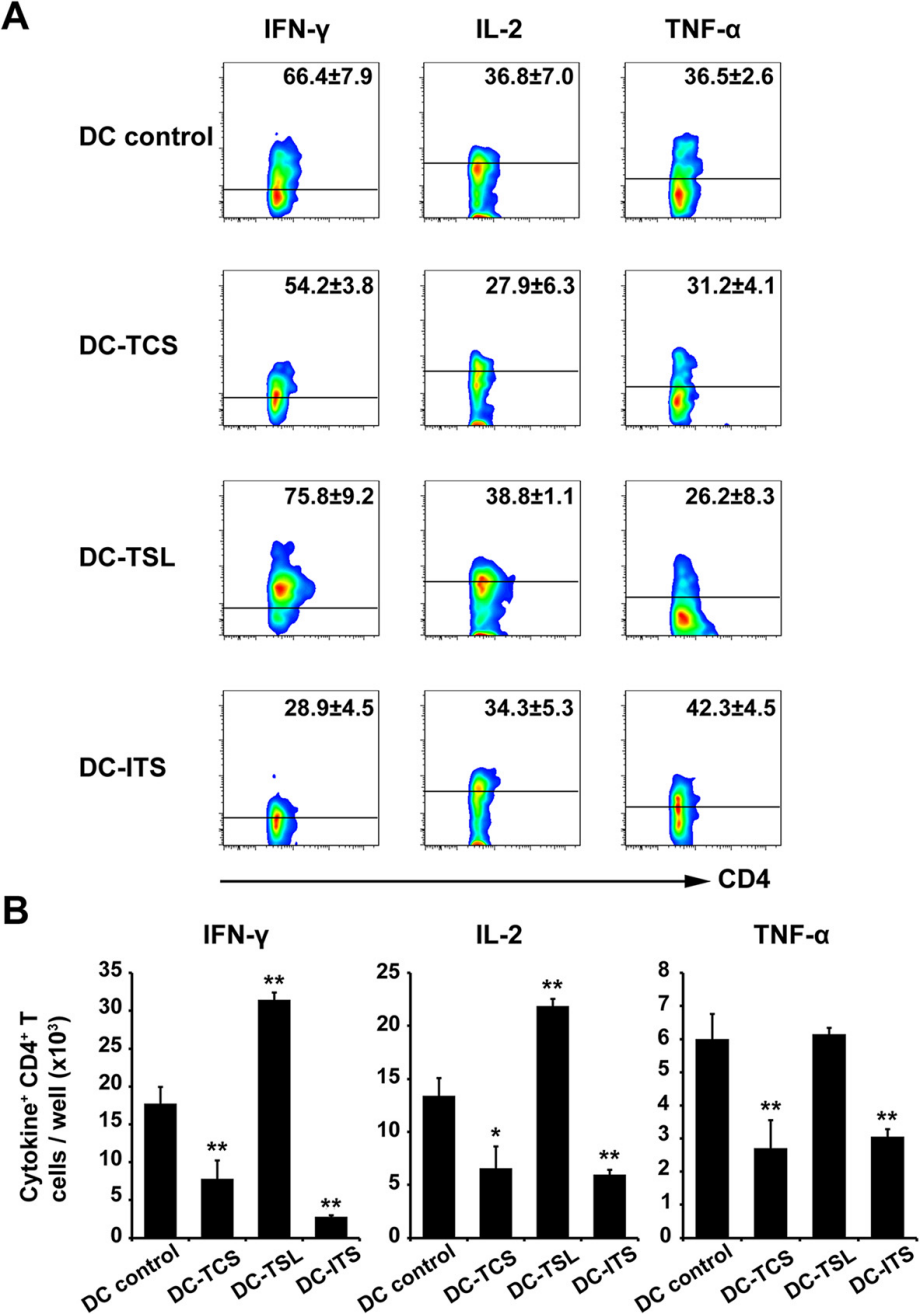
Induction of TAA-specific Th1 cytokines by three DC Ag-loading strategies. DCs were pulsed with either SNU899 tumor cell supernatant, stressed lysate, or irradiated tumor cells. Autologous PBLs were stimulated twice with Ag-loaded mature DCs. On day 6 after the second stimulation, production of IFN-γ, IL-2, and TNF-α by TAA-specific CD4^+^ T cells was detected by intracellular staining. **a** Numbers indicate the percentage of gated CD4^+^ T cells. When PBLs were stimulated by TCL-DCs, the percentage of IFN-γ and IL-2-secreting CD4^+^ T cells was higher than in other groups, whereas DC-ITC induced a higher percentage of TNF-α-secreting CD4^+^ T cells. **b** Number of Th1 cytokine (IFN-γ, IL-2, and TNF-α)-positive cells were assessed by flow cytometry. Compared with the DC control, TCL-DCs stimulated an effective TAA-specific T cell response, whereas DC-TCS and DC-ITC inhibited such responses. These results clearly demonstrate the efficacy of stressed lysis as a means of tumor cell preparation for DC loading. Data are mean values ± SD of triplicate determinations. Statistical comparisons were performed using Student’s t-test. *P < 0.05 vs. DC control; **P < 0.01 vs. DC control

Cytokines visualized by intracellular staining were used to confirm the effect of Ag priming on Th1 responses (Fig. 4). Reactive lymphocytes stimulated by Ag-loaded DC lysate showed a higher proportion of IFN-γ and IL-2-secreting CD4^+^ T cells than those stimulated by DC controls, but a lower ratio of TNF-α-secreting CD4^+^ T cells. DC-TCS reduced all Th1 cytokines (IFN-γ, IL-2, and TNF-α) secreted by CD4^+^ T cells. The irradiation strategy resulted in a decrease in the proportion of cells secreting IFN-γ and IL-2, but increased those secreting TNF-α (Fig. 4a). Furthermore, significant numbers of IFN-γ and IL-2-secreting CD4^+^ T cells were induced by DC-TSL (Fig. 4b), which could be attributed to the increased proportion of cytokine-secreting CD4^+^ T cells (Fig. 4a) and increased number of TAA-specific CD4^+^ T cells (Fig. 3a). However, TCS and ITC strategies inhibited the generation of Th1 cytokine-secreting CD4^+^ T cells.

These data suggest strongly that SNU899 cell-derived stressed lysate Ags induce a more efficient TAA-specific Th1 cytokine response, and may be the optimal Ag loading method.

### Induction of TAA-specific CTL responses

**Fig. 5:**
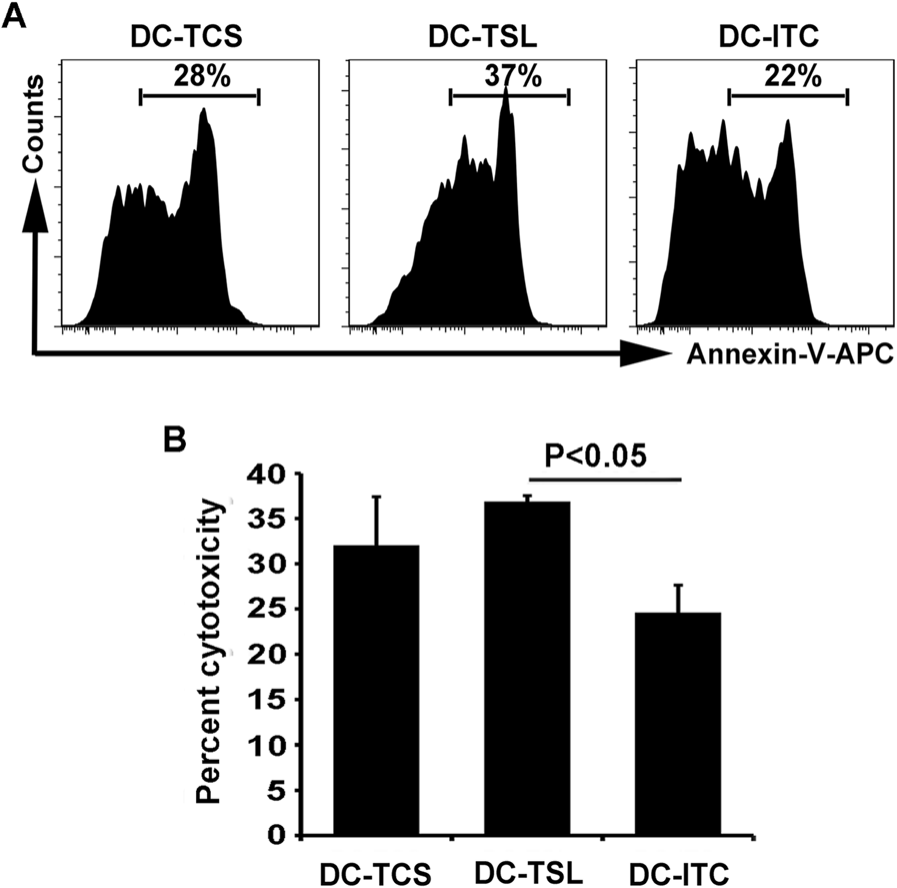
Induction of TAA-specific CTL activity by three DC Ag-loading strategies. **a** Ag-loaded DCs were used as stimulators of autologous PBLs to compare their efficacy in CTL stimulation against Ag-pulsed target cells. PBLs and DCs were generated from a single donor. Data are from one representative experiment. **b** TAA-specific cytotoxicity was determined by flow cytometry. Data are the mean percentages of cytotoxicity ± SD of triplicate determinations. TCL-DCs induced a more potent CTL response compared with DC-ITC as shown by the increased cytotoxicity from SNU899-derived Ag-pulsed autologous DCs used as targets at a 10:1 E:T. Statistical comparisons were performed using Student’s t-test

The three types of Ag-loaded DCs were used as stimulators for autologous PBLs to compare their efficacy for CTL stimulation. SNU899-derived lysate-pulsed immature DCs autologous to the CTLs were used as target cells for recognition of their MHC-antigen complexes by reactive CTLs. The ratio of apoptotic target cells was determined with flow cytometry by subtraction of basal level apoptosis. CTLs stimulated by DC-TSL resulted in a higher ratio of apoptotic target cells (Fig. 5a). Statistical analysis showed that DC-TSL were more effective at stimulating CTL responses than DC-TCS (36.9 ± 0.6 % and 32.1 ± 5.4 %, respectively; Fig. 5b). DC-ITC induced a much lower CTL response (24.6 ± 3.0 %; Fig. 5b) compared with DC-TSL (*P* = 0.03).

## Discussion

DC vaccines take advantage of the potent antigen-presenting capacity of DCs to stimulate primary and secondary T and B cell immune responses. Clinical trials have been performed for several advanced cancers such as melanoma and renal, prostate, and colon cancers, which have shown encouraging results [5–7]. DC vaccines targeting mutated proteins in the p53 pathway [22] and human papilloma virus peptides have been produced for the treatment of HNSCC [22, 23]. These TAA-loaded DCs stimulate immunological and clinical responses in certain patients. However, a significant proportion of patients remain unresponsive to immunomodulatory therapy.

Unlike specific TAAs, whole tumor cells or their derivatives may better apply to various tumors for which few or no defined tumor-specific antigens are available, such as for laryngeal cancer. Furthermore, whole tumor cell immunizations result in polyvalent stimulation of both CD4+ Th cells and CD8+ CTLs against a broad range of Ags. Therefore, DCs loaded with Ags in this manner may be used to treat laryngeal cancer.

Among the current methods for pulsing DCs with whole tumor-derived materials, tumor cell lysates, which are prepared by repeated cycles of freezing and thawing with solid debris removed by centrifugation, have been widely applied in early stage clinical trials. However, an increasing number of trials have shown that the immune response elicited by DC-TSL does not generate a sufficient antitumor potency [10, 13, 14]. Recently, several studies have demonstrated that freeze-thawed lysates suppress DC maturation and function in vitro, and are ineffective for loading DCs for therapies in vivo [15, 16]. Our preliminary study also suggests that DCs cultured in the presence of human laryngeal cancer-derived lysates decreased surface expression of B7–1, B7–2, and MHC molecules, and inhibit T cell proliferation (data not shown). Potential explanations for these findings include the use of different tumor stains, alternate vaccination schedules, and the use of DCs at various stages of maturity [10].

To elicit an effective immune response, we used three strategies for loading DCs with tumor-derived Ags and compared their ability to stimulate CTL activity. The loading strategies included using DC-TCS, DC-TSL, and DC-ITC. Our results show that human laryngeal cancer-derived TSL was an effective TAA source for pulsing DCs. DC-TSL not only induced the most potent CTL activity, but also induced the strongest MHC class I TAA-specific T response (proliferation of TAA-specific CD8^+^ T cells), the highest expansion of TAA-specific T cells (based on the number of TAA-specific T cells), and the strongest Th1 cytokine response (elevated levels of IFN-γ and IL-2 production). Interestingly, DC-TSL induced both class I and class II TAA-specific responses, suggesting that DC-TSL are capable of both class I and II cross-presentation. These results emphasize that the choice of Ag-loading strategy is critical to the strength of the immune response.

We identified alterations in the DC phenotype after treatment with tumor-derived Ags. DC-TSL expressed the highest level of HLA-DR and CD86, whereas DC-ITC also had elevated HLA-DR expression but expressed the lowest levels of co-stimulatory molecules (CD80, CD86, and CD40). HLA-DR expression on the surface of DC-TCS remained at the same level as that in DC controls. A significant body of evidence has shown that stimulating a stress response in tumor cells increases the production of HSPs, which may expand the repertoire of TAAs and enhance TAA delivery to professional antigen-presenting cells (APCs) [17, 24, 25]. Additionally, stress-induced HSPs stimulate DCs and induce APC cytokine and chemokine secretion [26–29]. Our findings support the idea that the stressed lysis strategy has advantages by providing a larger TAA repertoire. Moreover, elevation of co-stimulatory molecules such as CD86 may enhance DC capacity to prime T cell responses. In contrast, cell culture supernatants may not contain sufficient or adequate Ags secreted by laryngeal cancer cells. Although one study reported that DC antigen presenting function can be improved by supernatants from retinoblastoma cells [18], it is not surprising that laryngeal cancer has a distinct tumor milieu to interact with DCs. LSCC cells may evade the host immune system through manipulation of their own immunogenicity, production of immunosuppressive mediators, and promotion of immunomodulatory cell types [30–32]. Consistent with some studies [33], there was a reduction of CD80, CD86, and CD40 in DC-ITC. Various mechanisms through which intact tumor cells suppress DC functions have been described, including cytokines (e.g., TGF-β, IL-10, and vascular endothelial growth factor), ceramide [34], and other tumor-derived lipids [35]. However, a complete understanding of the mechanisms by which DC suppression functions will require additional study.

Studies have shown that CD8^+^ and Th1 cells play an important role in controlling tumor growth. For example, CD8^+^ T cells mediate antitumor immunity [36]. Th1 cells, a subset of CD4^+^ T cells, constitutively express IFN-γ and TNF-α, and play a role in priming tumor-specific CTLs through the release of soluble IL-2 in the proximity of CTLs [37]. In addition, induction of MHC class I tumor-specific immunity requires epitope linkage between Th1 and CTL epitopes, which is important for CTL induction [38]. In the current study, DC-TSL pulsing was the only effective method to prime CD8^+^ and CD4^+^ T cells. This result indicates that a TSL is not only processed for MHC class I presentation, but also cross-presented by the MHC class II pathway, consistent with the elevation of class II molecules (HLA-DR) on DC-TSL. Further experimentation showed that both the percentage and number of Th1 cells (including IFN-γ- and IL-2-secreting cells) were increased in T cell and DC-TSL co-culture, which strongly supports the choice of TSL-pulsed as a promising CTL activator against laryngeal cancer. It is worth noting that the stronger and broader T cell response induced by DC-TSL may benefit from production of HSPs, such as HSP70 and HSP90 [39, 40], whose antitumor activity is exerted through various mechanisms. The inefficiency of DC-ITC to induce Ag-specific T cell responses can be explained by the inefficient cross-presentation of TAAs by DCs that express low levels of co-stimulatory molecules. Although apoptotic tumor cells induced by irradiation effectively prime APCs in vitro, they likely cannot stimulate DCs to generate an antitumor immune response in the absence of additional maturation [41].

The incompetence of DC-TCS, which express low levels of MHC molecules (e.g., HLA-DR) and maturation markers (e.g., CD83), may result from inhibition by the tumor milieu and fewer laryngeal cancer cell-secreted Ags. Although CTL activity of DC-TCS was lower than that of DC-TSL, which induced the highest as expected, there was no significant difference between them. The increased CTL activity of DC-TCS-stimulated PBLs may be due to more active natural killer and natural killer T cells, which comprise the majority of non-CD8 T lymphocyte effector cells [42]. Additionally, to ensure that the Ag-MHC complex was recognized by CTLs, we used Ag-pulsed DCs as the source of MHC-matched target cells, which may not accurately reflect tumor cell susceptibility to CTL lysis [43].

## Conclusions

We demonstrate that a heat-treated TSL is an effective source of TAAs for pulsing DCs to treat human LSCC. DC-TSL induced the greatest expansion of TAA-specific T cells, the strongest Th1 cytokine response, and the most potent CTL activity, whereas DC-TCS or DC-ITC inhibited T cell activation and induced only a certain extent of CTL activity. Although the efficacy of this loading strategy needs to be tested further, such as in a setting whereby the CTL response could be tested using autologous laryngeal cancer cells, we have provided an encouraging alternative strategy for DC-based immunotherapy for laryngeal cancer.

## Abbreviations

DCs: dendritic cells
TAAs: tumor-associated antigens
CTL: cytotoxic T lymphocyte
HNSCC: head and neck squamous cell carcinoma
LSCC: laryngeal squamous cell carcinoma
Ag: antigen
HSPs: heat shock proteins
MHCs: major histocompatibility complexes
TSL: tumor-stressed lysate
PBMCs: peripheral blood mononuclear cells
IL: interleukin
GM-CSF: granulocyte macrophage-colony stimulating factor
LPS: lipopolysaccharide
PBLs: peripheral blood lymphocytes
SD: standard deviation
APCs: antigen-presenting cells. DC-TCS: DCs pulsed with the tumor cell supernatant
DC-TSL: DCs pulsed with the whole-cell tumor stressed lysate
DC-ITC: DCs pulsed with irradiated tumor cells
